# Characterization of 405B8H3(D‐E), a newly engineered high affinity chimeric LAG‐3 antibody with potent antitumor activity

**DOI:** 10.1002/2211-5463.13648

**Published:** 2023-06-11

**Authors:** Xiaoxuan Lan, Teddy Tat Chi Yang, Yinghui Wang, Baoyuan Qu, Shaofeng Rong, Ningning Song

**Affiliations:** ^1^ School of Perfume and Aroma Technology Shanghai Institute of Technology China; ^2^ Shanghai ChemPartner Co., Ltd. China; ^3^ Jiangsu Huaiyu Pharmaceutical Co., Ltd. China

**Keywords:** 405B8H3(D‐E), antibody, cancer, HEK293, immunotherapy, LAG‐3

## Abstract

Lymphocyte activation gene‐3 (LAG‐3) is a type I transmembrane protein with structural similarities to CD4. Overexpression of LAG‐3 enables cancer cells to escape immune surveillance, while its blockade reinvigorates exhausted T cells and strengthens anti‐infection immunity. Blockade of LAG‐3 may have antitumor effects. Here, we generated a novel anti‐LAG‐3 chimeric antibody, 405B8H3(D‐E), through hybridoma technology from monoclonal antibodies produced in mice. The heavy‐chain variable region of the selected mouse antibody was grafted onto a human IgG4 scaffold, while a modified light‐chain variable region was coupled to the human kappa light‐chain constant region. 405B8H3(D‐E) could effectively bind LAG‐3‐expressing HEK293 cells. Moreover, it could bind cynomolgus monkey (cyno) LAG‐3 expressed on HEK293 cells with a higher affinity than the reference anti‐LAG‐3 antibody BMS‐986016. Furthermore, 405B8H3(D‐E) promoted interleukin‐2 secretion and was able to block the interactions of LAG‐3 with liver sinusoidal endothelial cell lectin and major histocompatibility complex II molecules. Finally, 405B8H3(D‐E) combined with anti‐mPD‐1‐antibody showed effective therapeutic potential in the MC38 tumor mouse model. Therefore, 405B8H3(D‐E) is likely to be a promising candidate therapeutic antibody for immunotherapy.

## Graphical abstract

Overexpression of lymphocyte activation gene‐3 (LAG‐3) enables cancer cells to escape immune surveillance, while its blockade reinvigorates exhausted T cells and strengthens anti‐infection immunity. Blockade of LAG‐3 may provide efficient antitumor treatment. LAG‐3 inhibition resulted in enhanced T‐cell activation as measured by increased interleukin‐2 secretion.

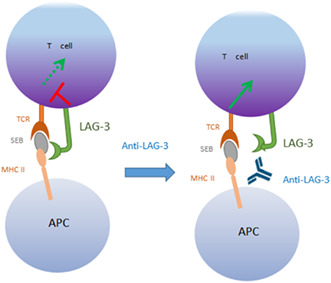

**Abbreviations**
CDRs: complementarity‐determining regions
cyno: cynomolgus monkey
EC50: half maximal effective concentration
ELISA: enzyme‐linked immunosorbent assay
FACS: fluorescence‐activated cell sorting
HPLC/SEC: high‐performance liquid chromatography/size exclusion chromatography
IACUC: Institutional Animal Care and Use Committee
IC50: half maximal inhibitory concentration
IL‐2: interleukin‐2
*K*_D_
: dissociation constant
LAG‐3: lymphocyte activation gene‐3
LSECtin: liver sinusoidal endothelial cell lectin
mAb: mouse monoclonal antibody
MHC‐II: major histocompatibility complex II
NK: natural killer cells
PBMC: human peripheral blood mononuclear cells
SEB: staphylococcal enterotoxin B
TCR: T‐cell receptor
Tregs: regulatory T cells
VH: variable heavy‐chain
VL: variable light‐chain

Triebel *et al*. [1] first discovered lymphocyte activation gene‐3 (LAG‐3) among molecules differentially expressed in interleukin‐2 (IL‐2)‐dependent natural killer cells (NK). LAG‐3 is composed of 525 amino acids, with four Ig‐like domains, termed domain 1 (D1) to domain 4 (D4). The D1 region contains a proline‐rich ring structure, which specifically binds to major histocompatibility complex II (MHC‐II) molecules [2]. Like CD4, LAG‐3 binds the MHC‐II on antigen‐presenting cells, but with a much stronger affinity, which effectively inhibit T‐cell activation [2]. LAG‐3 is expressed on CD4+ and CD8+ T cells, regulatory T cells (Tregs) [3, 4], NK cells [5] and dendritic cells [6]. LAG‐3 is also expressed by exhausted CD4+ and CD8+ tumor‐infiltrating T cells [7, 8, 9, 10, 11], and by Tregs in peripheral blood and tumor tissues of patients with melanoma and non‐small‐cell lung cancer [12, 13]. Liver sinusoidal endothelial cell lectin (LSECtin) is another known ligand of LAG‐3. In the melanoma microenvironment, the binding of LSECtin and LAG‐3 molecules can suppress the secretion of interferon‐γ from antigen‐specific T cells, which inhibits their antitumor activity [14, 15]. Hence, blocking the interactions between LAG‐3 and its potential ligands may be an effective strategy for cancer immunotherapy. In mouse models of head and neck squamous cell carcinoma, anti‐LAG‐3 antibodies have been shown to delay tumor growth [16]. Recently, LAG‐3 has emerged as a potential immune checkpoint molecule for cancer therapy, which has been evaluated in 108 interventional clinical trials involving anti‐LAG‐3 monoclonal antibodies, (LAG‐3/PD‐1) bispecific antibodies, or LAG‐3 fusion proteins [17]. For instance, the fusion protein IMP321 was tested for its ability to improve antitumor immune responses (NCT03252938; www.clinicaltrials.gov); the LAG‐3/PD‐1 bispecific antibody RO7247669 is currently tested in patients with advanced and/or metastatic solid tumors (NCT04140500; www.clinicaltrials.gov). In 2022, the combined therapy Opdualag, which targets PD‐1 (nivolumab) and LAG‐3 (relatlimab‐rmbw), was tested for unresectable or metastatic melanoma and established a significant milestone for cancer therapies [18]. Therefore, a safe and highly specific anti‐LAG‐3 antibody is highly desirable either for monotherapy or as an option for combined therapy.

Based on hybridoma technology, we generated and characterized a novel chimeric anti‐LAG‐3 antibody that we called 405B8H3(D‐E). *In vitro*, the ability of 405B8H3(D‐E) to bind cynomolgus monkey (cyno) LAG‐3 protein on the cell surface was stronger than that of the reference antibody BMS‐986016, a human anti‐LAG‐3 IgG4 antibody previously tested against solid tumors and hematological malignancies in clinical trials (ClinicalTrials.gov Identifier: NCT02061761). This result suggests that 405B8H3(D‐E) could be tested *in vivo* for preclinical toxicology and pharmacokinetics studies on primates. 405B8H3(D‐E) induced secretion of IL‐2 and could block the interactions between LAG‐3 and LSECtin and MHC‐II molecules. 405B8H3(D‐E) in combination with an anti‐mouse PD‐1 antibody also exhibited an antitumor effect in a MC‐38 murine colon carcinoma model generated in human LAG‐3 (hLAG‐3) knock‐in mice. Therefore, 405B8H3(D‐E) is a novel anti‐LAG‐3 antibody with good potential for immunotherapy.

## Materials and methods

### Recombinant protein (hLAG‐3ECD‐hFc)

The DNA fragment corresponding to the amino acid sequence 23–450 (UniProtKB: P18627, L23‐L450) of the extracellular region (ECD) of human LAG‐3 was fused to a sequence encoding a human IgG Fc tag and cloned into a pCPC vector (Invitrogen, Carlsbad, CA, USA; #V044‐50). This vector was transfected transiently into HEK293 cells (Invitrogen) to produce the corresponding fusion protein (hLAG‐3ECD‐hFc). Protein A affinity chromatography Sepharose FF (GE Healthcare, Fairfield, CT, USA; #17‐1279‐03) and Superdex 200 (GE Healthcare, Chicago, IL, USA) size exclusive chromatography were used to purify the recombinant proteins and antibodies from the supernatant. SDS/PAGE and high‐performance liquid chromatography/size exclusion chromatography (HPLC/SEC) were used to determine the molecular weight and purity of the target protein.

### Recombinant protein (BMS‐986016 mAb)

BMS‐986016, initially invented by Bristol‐Myers Squib™, was generated in the laboratory according to publicly available sequences. The amino acid sequence of BMS‐986016 was derived from the patent (WO2015116539), encoding the constant region of the human IgG4 heavy chain and encoding the constant region of the human kappa light chain was cloned into pCPC vector followed by transiently transfecting HEK293 cells (Invitrogen) free bovine serum for reference protein production. Protein A affinity chromatography Sepharose FF (GE Healthcare, Fairfield, CT, USA; #17‐1279‐03) and Superdex 200 (GE Healthcare, Chicago, IL, USA) size exclusive chromatography were used to purify the recombinant proteins and antibodies from the supernatant.

### HEK293 stable cell lines

#### HEK293/hLAG‐3 and HEK293/cynoLAG‐3

The nucleotide sequence encoding full‐length human LAG‐3 was cloned into the pLVX‐IRES vector (Clontech, Mountain View, CA, USA), which was subsequently transfected into HEK293 cells to obtain a stable HEK293/hLAG‐3 cell line. The nucleotide sequence encoding full‐length cyno LAG‐3 was cloned into the pIRES vector (Clontech), which was subsequently transfected into HEK293 cells to obtain a stable HEK293/cynoLAG‐3. The stable HEK293/hLAG‐3 and HEK293/cynoLAG‐3 cell lines were cultured in selecting Dulbecco's modified minimal essential medium (DMEM; Corning, Corning, NY, USA; #10‐013‐CVR) containing 0.5 μg·mL^−1^ puromycin (Gibco, Grand Island, NY, USA; #A1113803) and 10% fetal bovine serum (FBS; Biological Industries, Beit Haemek, Israel) for 2 weeks.

#### Peripheral blood mononuclear cell

Human peripheral blood mononuclear cells (PBMCs) were isolated from whole blood using Ficoll‐Paque (GE Healthcare) density gradients followed by centrifugation at 400 ***g*** for 30 min. This study was conducted in compliance with the guidelines set by the Declaration of Helsinki. The use of the human materials analyzed in this study was approved by the Institutional Ethics Committee (IEC) of ChemPartner (Shanghai, China; IEC protocol no.: IEC001‐R2015), and a written informed consent was provided by all donors.

#### Generation of murine antibodies

SJL and Balb/c mice (Shanghai SLAC, Shanghai, China) aged 6–8 weeks were immunized with purified hLAG‐3ECD‐hFc. For the first immunization, the mice were injected with 50 μg of immunogen and 0.25 mL of complete Freund's adjuvant (Sigma, St. Louis, MO, USA; #F5881). Two weeks later, the mice were injected with 50 μg of immunogen and 0.25 mL of incomplete Freund's adjuvant (Sigma; #F5506) to enhance the immune response, followed by a third immunization 3 weeks later with incomplete Freund's adjuvant. One week after each booster immunization, blood samples were collected, and the serum antibody titers were analyzed by enzyme‐linked immunosorbent assay (ELISA) using hLAG‐3ECD‐hFc as coating antigen. When the serum had reached a sufficient antibody titer, the immunized mouse spleen cells and mouse SP2/0 myeloma cells (ATCC; Manassas, VA, USA; C # CRL‐1581) were isolated with high purity and mixed at 5 : 1 ratio for electrofusion to prepare hybridomas. The resulting hybridomas were screened for specificity by analysis of their supernatants by ELISA, using the hLAG‐3ECD‐hFc protein as coating antigen, and by fluorescence‐activated cell sorting (FACS), using HEK293/hLAG‐3 cells as positive target cells. The generation of murine antibodies was approved by the institutional ethics committee (#A998AL0034).

#### Immunoglobulin variable region cloning and sequencing

For initial RNA isolation, the culture supernatant of the selected clones was tested for antigen binding, and an aliquot containing 5 × 10^7^ hybridoma cells was harvested by centrifugation at 500 ***g***. Total cDNA synthesis was performed using Prime Script RT Master Mix RR036 (Takara, Kyoto, Japan) following the manufacturer's instructions. Next, the cDNAs encoding the variable regions of the immunoglobulins produced by the hybridoma were cloned into a TA vector. The DNA sequences corresponding to the mouse monoclonal antibody (mAb) variable regions were analyzed by DNA sequencing after PCR amplification, as described previously [19]. The PCR products (5 μL) were checked by agarose gel electrophoresis and then recovered and purified using the NucleoSpin Gel & PCR Clean‐up kit, according to the manufacturer's instructions (#740609; Macherey‐Nagel, Düren, NRW, Germany). After a PCR reaction, the positive samples were sent for sequencing. This strategy led to the sequence identification of the variable regions of six candidate clones.

### Preparation of human‐mouse IgG4 chimeric anti‐LAG‐3 monoclonal antibodies

#### Hotspots mutation

Following amplification and sequencing, the cDNA corresponding to the variable regions of the heavy and light chains of the anti‐LAG‐3 antibodies from the mouse hybridoma was obtained. The sequence was analyzed to identify major hot spot sites including unpaired cysteine residues, N‐glycosylation site, and deamination site within the complementarity‐determining regions (CDRs) [20, 21]. In this study, the heavy‐chain variable (VH) region/light‐chain variable (VL) region CDR residues were determined and annotated with Kabat numbering system. According to protein sequences, the Asp‐Gly (DG) motif laying in the CDR2 of the murine anti‐LAG‐3 antibody light chain may be involved in the interaction with the antigen [22, 23]. Therefore, to conserve its functionality, this motif was mutated into amino acids with similar structures and properties: Since serine (S) is similar to aspartic acid (D) and glutamate (E), DG was mutated to SG or EG.

#### Expression and purification

The mouse VH gene fragments were cloned into pCPC vectors containing a signal peptide and a cDNA encoding the constant region of the human IgG4 heavy chain. The modified mouse light‐chain variable gene corresponding to the same hybridoma was cloned into a pCPC vector comprising a signal peptide and a cDNA encoding the constant region of the human kappa light chain. The heavy‐ and light‐chain expression vectors were co‐transfected into FreeStyle 293‐F cells (1.5 × 10^6^ cells·mL^−1^) and cultured for 1 week. Finally, the resulting chimeric antibody was purified using Protein A.

The DG motif was mutated to SG or EG according to protein sequences. The SG and EG mutated variants were grafted onto chimeric antibodies, which were selected according to their EC50 values for the binding of HEK/hLAG‐3 and HEK/cyno293 stably transformed cell lines. The SG mutated variant antibody was named 405B8H3(D‐S), and EG mutated variant antibody was named 405B8H3(D‐E). Because 405B8H3(D‐E) had lower EC50 values, we selected it for further testing.

#### Assessment of binding activity by flow cytometry

For the cell surface antigen‐binding assay, HEK293/hLAG‐3 cells or HEK293/cyno hLAG‐3 cells, HEK293 cells not expressing LAG‐3(HEK293‐Blank) were adjusted to 2 × 10^5^ cells per well of a 96‐well plate. Three anti‐LAG‐3 antibodies or isotype control (0–200 nm) were added to the cells and incubated at 4 °C for 1 h. After removal of the primary antibodies by centrifugation at 500 ***g*** for 5 min, a goat anti‐human IgG (Fab fragment) Secondary Antibody Alexa Fluor® 488 conjugate (Jackson; West Grove, PA, USA #109‐545‐006) was added to the cells and incubated at 4 °C for 1 h before washing twice in PBS containing 2% FBS. The analysis was performed using a FACS Canto II flow cytometer (BD Biosciences, Franklin Lakes, NJ, USA). Nonlinear regression analysis was performed using graphpad prism 6 (GraphPad Software, San Diego, CA, USA). The half maximal effective concentration (EC50) values were calculated from the mean fluorescence intensity of the stained cells obtained with different primary antibody concentrations, reflecting the binding avidity of the antibody.

#### Binding assessment by ELISA

The antigens (hLAG‐3ECD‐hFc, 1 μg·mL^−1^, 100 μL/well) were coated onto 96‐well ELISA plates (BEAVER; Suzhou, Jiangsu, China #40301) by overnight incubation at 4 °C. The next day, the plates were blocked with 1% BSA (AMRESCO; Solon, OH, USA #0332‐1KG) in PBS containing 0.05% Tween‐20 (PBS‐T) for 2 h at 37 °C. Then, each of the two LAG‐3 antibodies (from 0 to 66 nm) was incubated in a volume of 100 μL per well for 2 h at 37 °C. The HRP‐labeled secondary antibody was added and incubated at 37 °C for 2 h. Between incubations, the plates were washed twice in PBS‐T. The HRP substrate TMB (Innoreagents; Huzhou, Zhejiang, China #TMB‐S‐003) was added at 100 μL/well for 30 min, and the quenching of the reaction was performed by adding 1 m HCl (100 μL/well) at room temperature. The absorbance was measured using a Molecular Devices SpectraMax Plus, Silicon Valley, CA, USA 384 at 450 nm.

#### Measurement of antibody avidity by Biacore measurement

The avidity of the candidate anti‐LAG‐3 mAbs was evaluated by measuring their binding kinetics in a label‐free bio‐layer interferometry assay. The anti‐human FC IgG was loaded onto the surface of the CM5 chip from 6000 to 10 000 RU, and FC1 was used as the reference channel. The anti‐LAG‐3 candidate mAbs were diluted to 5 μg·mL^−1^ in HBS‐EP and 10 mm NaAc buffer with 10 μL·min^−1^ to obtain a response value of 100–300 RU. A His tag antigen protein was diluted to 100 nm at 30 μL·min^−1^ across the surface of the chip by Octet Red 384 (ForteBio, Fremont, CA, USA). The dissociation constant (*K*_D_) was calculated using the *K*_D_ = kd·ka^−1^ formula.

#### LAG‐3/MHC‐II blocking assay by flow cytometry

Raji cells expressing MHC‐II were adjusted to a concentration of 2 × 10^6^ cells·mL^−1^, seeded onto a 96‐well plate (Corning; #3799; 100 μL/well), and incubated at 4 °C for 30 min. Various concentrations of anti‐LAG‐3 antibodies or isotype control (from 0 to 200 nm) were mixed with hLAG‐3ECD‐hFc (1 μg·mL^−1^) in a total volume of 100 μL/well and incubated at room temperature for 30 min. Following incubation, the antibodies were added to the harvested Raji cells, incubated at 4 °C for 2 h, and incubated with a goat anti‐human IgG (Fab fragment) Secondary Antibody Alexa Fluor® 488 conjugate (Jackson; #109‐545‐006) for 1 h at 4 °C. The cells were washed twice in PBS containing 2% FBS. The analysis was performed on a FACS Canto II flow cytometer (BD Biosciences). The half maximal inhibitory concentration (IC50) values were calculated using graphpad prism version 6 to evaluate the LAG‐3/MHC‐II blocking activity of the antibody.

#### MHC‐II(Raji) binding assay by flow cytometry

Raji‐MHC‐II cells were seeded onto a 96‐well plate and incubated with hLAG‐3ECD‐hFc or hFc proteins. A secondary Alexa Fluor 488‐conjugated antibody was used for detection by flow cytometry. The mean fluorescence intensity is shown as the mean ± SEM, calculated from experimental duplicates. The analysis was performed on a FACS Canto II flow cytometer (BD Biosciences). The blocking activity of the antibody on Raji‐MHC‐II cells was evaluated by calculating the mean fluorescence intensity of the stained cells using graphpad prism version 6.

#### LAG‐3/LSECtin blocking assay by ELISA

The antigens (hLAG‐3ECD‐hFc, 1 μg·mL^−1^, 100 μL/well) were coated onto 96‐well ELISA plates (BEAVER; #40301) by overnight incubation at 4 °C. The next day, the plates were blocked with 1% BSA (AMRESCO; #0332‐1KG) in PBS containing 0.05% Tween‐20 (PBS‐T) for 2 h at 37 °C. Then, each of the two LAG‐3 antibodies (from 0 to 200 nm) was incubated in a volume of 50 μL per well with the His tag LSECtin ligand (1 μg·mL^−1^; R&D System, Minneapolis, MN, USA; #2947‐CL) for 2 h at 37 °C. The HRP‐labeled anti‐His tag antibody (GenScript; NanJing, Jiangsu, China #A00612) was added and incubated at 37 °C for 2 h. Between incubations, the plates were washed twice in PBS‐T. The HRP substrate TMB (Innoreagents; #TMB‐S‐003) was added at 100 μL/well for 30 min, and the quenching of the reaction was performed by adding 1 m HCl (100 μL/well) at room temperature. The absorbance was measured using a Molecular Devices SpectraMax Plus 384 at 450 nm.

#### PBMC‐staphylococcal enterotoxin B assay by ELISA

Human peripheral blood mononuclear cells were isolated from a Ficoll‐Paque Plus density gradient, plated at 1 × 10^5^ cells (100 μL/well) into 96‐well plates (Corning; #3599), mixed with the candidate LAG‐3 antibodies at concentrations ranging from 10 to 0.016 μg·mL^−1^, and incubated at room temperature for 30 min. Staphylococcal enterotoxin B (SEB) superantigen (50 μL at 100 ng·mL^−1^; Beijing Kangbobeining Company, Haidian District, Beijing, China) was added to the reaction wells and incubated at 37 °C with 5% CO_2_. After 3 days, the IL‐2 secretion levels were determined by ELISA (R&D System kit; IL‐2 DoSet # DY202) according to the manufacturer's instructions. The absorbance was measured with a Molecular Devices SpectraMax Plus 384, at 450 nm.

#### MC38 tumor model in hLAG‐3 knock‐in mice

All experiments involving animals in this study were approved by Biocytogen Animal Care Committee protocol number X998HL0074. The murine colon carcinoma cell line MC‐38 was acquired from the National Cancer Institute (Rockville, MD, USA). MC38 cells (1 × 10^7^ cells in DMEM) were implanted subcutaneously into the right flank of hLAG‐3 knock‐in C57BL/6 mice (6‐week‐old; Biocytogen, Beijing, China). The experimental mice were randomly divided into three groups (*n* = 10 per group). Mice with tumor sizes of approximately 50–80 mm^3^ were enrolled into the efficacy study. Administration started from the day of randomization (defined as D0) and all the tested reagents were administered at 0.1 mL·10 g^−1^ based on the animal body weight on the day of dosing. Mice were treated as follows: Rat IgG2a 10 mg·kg^−1^ + hIgG4 10 mg·kg^−1^; anti‐mPD‐1‐antibody (Bio X Cell; West Lebanon, NH, USA # BE0146) 10 mg·kg^−1^ + hIgG4 10 mg·kg^−1^; 405B8H3(D‐E) 10 mg·kg^−1^ + anti‐mPD‐1‐antibody 10 mg·kg^−1^, injected intraperitoneally on D0, 3, 7, and 11. The mice were monitored daily, and the body weight was recorded on the working days. Tumor size was measured three times per week during the treatment. From D11, the animals started to reach a tumor volume of 2000 mm^3^ and were euthanized individually. The entire study was terminated on D28 as the last mouse reached the endpoint. This study was conducted following protocols that were reviewed and approved by the Institutional Animal Care and Use Committee (IACUC). After tumor inoculation, the animals were checked daily for morbidity and mortality. The data related to body weight, tumor growth, and the time‐to‐endpoint Kaplan–Meier survival curve were plotted using graphpad prism 6. The differences in tumor growth between groups were analyzed using two‐way RM ANOVA. The survival data were analyzed with log‐rank test. A *P*‐value < 0.05 was considered statistically significant.

## Results

### Generation of the anti‐hLAG‐3 antibody 405B8H3(D‐E)

A hLAG‐3ECD‐hFc fusion protein was used as immunogen in Balb/c and SJL mice to produce mouse anti‐human LAG‐3 antibodies through hybridoma technology. The gene fragments encoding the variable regions of the heavy and light chains of the antibodies produced by the resulting hybridomas were subcloned using conventional biomolecular methods. Fusion protein genes were then constructed by cloning the cDNAs encoding the VH regions in frame with a cDNA encoding a human IgG4 (Ser228Pro) constant region, which recruits fewer Fc‐dependent effector functions compared with other human IgG subclasses [24, 25, 26]. Similarly, the cDNAs encoding the VL regions, in which the aspartic acid D at position 56 was mutated to a glutamate E, were cloned in frame with a cDNA encoding the human kappa constant region. One of the highly specific positive clones was selected and named 405B8H3(D‐E). This antibody was further characterized for its binding properties using the HEK293/hLAG‐3 and HEK293/cynoLAG‐3 cell lines, expressing, respectively, the human and cynomolgus monkey LAG‐3 protein, and for its blocking activity against its two ligand MHC‐II and LSECtin. Since the sequence of the variable region of 405B8H3(D‐E) differs from those of existing antibodies, with, for example, homology < 92% with BMS‐980616 sequence, 405B8H3(D‐E) can be considered a novel antibody.

### Assessment of the binding properties of 405B8H3(D‐E) by FACS, ELISA, and Biacore

**Fig. 1:**
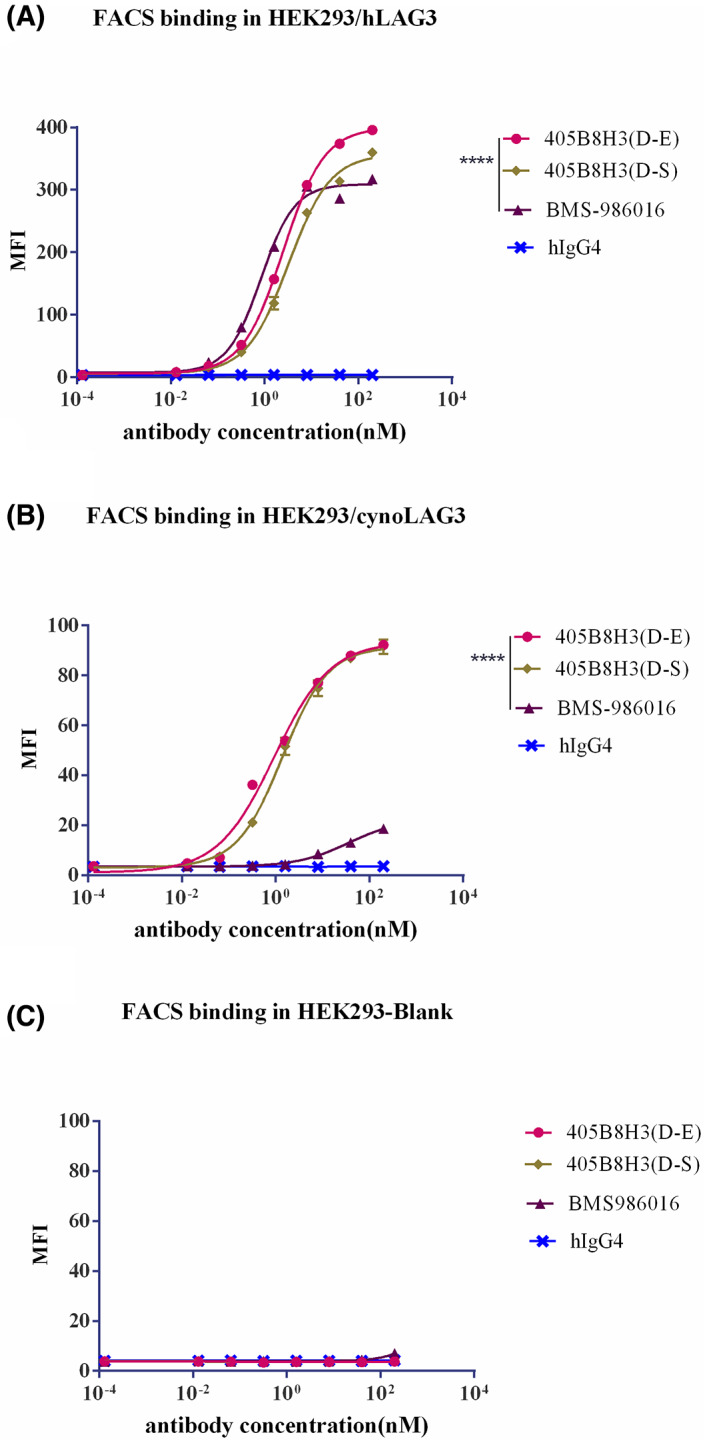
Characterization of 405B8H3(D‐E) *in vitro*. (A, B) Flow cytometric analysis of the mean fluorescence intensity of (A) HEK293/hLAG3 cells or (B) HEK293/cynoLAG3 cells stained with different concentrations of 405B8H3(D‐E), 405B8H3(D‐S) or BMS‐986016 anti‐LAG‐3 antibodies, or a negative control (hIgG4), and detected with an Alexa Fluor 488‐conjugated anti‐human secondary antibody. The experiment was performed three times in which it gave similar results. The mean fluorescence intensity is shown as means ± SEM calculated from experimental duplicates (A, B). 405B8H3(D‐E) did not bind to HEK293 cells not expressing LAG‐3 (Fig. 1C). The EC50 values of the indicated antibodies were calculated using graphpad prism, version 6; ****P < 0.0001, obtained by using Two‐way ANOVA analysis.

**Fig. 2:**
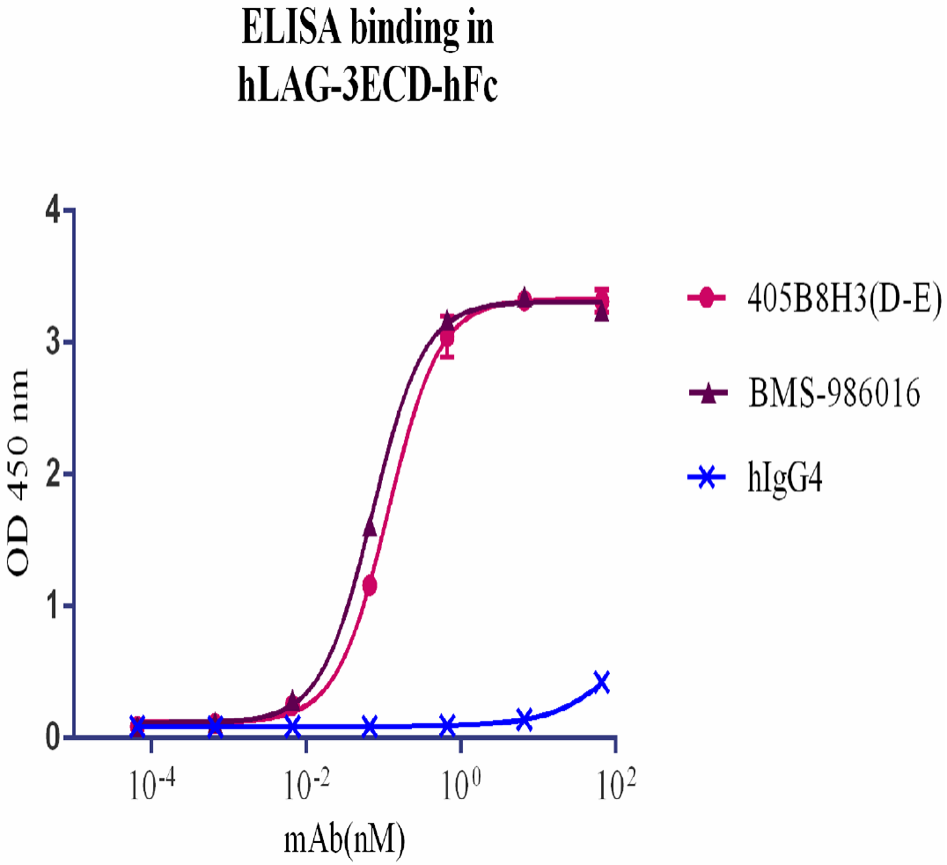
Characterization of 405B8H3(D‐E) *in vitro*. Analysis of 405B8H3(D‐E) and BMS‐986016 anti‐LAG‐3 antibodies' avidity by ELISA. Different concentration of the antibodies or of a negative control antibody (hIgG4) were tested against recombinant LAG‐3 proteins and revealed with an HRP‐labeled secondary antibody. The absorbance OD450 resulting from the enzymatic activity, which reflects the avidity of the anti‐LAG‐3 antibodies for LAG‐3, was measured at 450 nm. Three independent experiments were performed, which gave similar results. The OD450 is shown as means ± SEM calculated from experimental duplicates. The EC50 values of the indicated antibodies were calculated using graphpad prism, version 6.

The EC50 values of 405B8H3(D‐S), 405B8H3(D‐E), and the previously characterized anti‐hLAG‐3 antibody BMS‐986016 were determined and compared by flow cytometry. To this, HEK293/hLAG‐3 and HEK293/cynoLAG‐3 cells were stained with the three different anti‐LAG‐3 antibodies. On HEK293/hLAG‐3 cells, the EC50 value of BMS‐986016 (EC50 = 0.84 nm) was lower than that of 405B8H3(D‐E) (EC50 = 2.51 nm) and 405B8H3(D‐S) (EC50 = 3.24 nm; Fig. 1A), while the maximum value of 405B8H3(D‐E) (maximum = 399.80 nm) was superior to that of BMS‐986016 (maximum = 309.30 nm) and 405B8H3(D‐S) (maximum = 357.1 nm). On HEK293/cynoLAG‐3 cells, the EC50 and maximal values of 405B8H3(D‐E) (EC50 = 0.92 nm, maximum = 93.48 nm) and 405B8H3(D‐S) (EC50 = 1.39 nm, maximum = 91.51 nm) superior to those of BMS‐986016 (EC50 = 36.27 nm, maximum = 22.54 nm) (Fig. 1B). We also took flow cytometry analysis to demonstrate that 405B8H3(D‐E) binds to HEK293 cells not expressing LAG‐3. 405B8H3(D‐E) did not bind to HEK293 cells not expressing LAG‐3 (Fig. 1C). Next, we evaluated the ability of 405B8H3(D‐E) to bind hLAG‐3ECD‐hFc effectively by ELISA. The EC50 and maximum values of BMS‐986016 (EC50 = 0.07 nm, maximum = 3.31 nm) were similar to those of 405B8H3(D‐E) (EC50 = 0.11 nm, maximum =3.32 nm; Fig. 2). Lastly, 405B8H3(D‐E) affinity was measured on a Biacore. 405B8H3 (D‐E) bound to recombinant hLAG‐3‐ECD antigen with a *K*_D_ of 2.50 nm, which was inferior to that of BMS‐986016 (1.49 nm; Table 1). 405B8H3(D‐E) demonstrated a cross‐binding reactivity with HEK293/cynoLAG‐3 cells, which makes it usable in primates for preclinical toxicology and pharmacokinetic studies *in vivo*.

**Table 1:** Representative data obtained with 405B8H3(D‐E) and reference antibodies.

	Affinity *K*_D_ (nm)	ELISA		FACS		FACS		LAG‐3/MHC‐II	LAG‐3/LSECtin
		hLAG‐3 ECD‐hFc		HEK293/hLAG‐3		HEK293/cynoLAG‐3			
		Maximum (index)	EC50 (nm)	Maximum (index)	EC50 (nm)	Maximum (index)	EC50 (nm)	IC50 (nm)	IC50 (nm)
405B8H3(D‐E)	2.50	3.32	0.11	399.80	2.51	93.48	22.54	2.55	0.20
BMS‐980616	1.49	3.31	0.07	309.30	0.84	0.92	36.27	2.66	0.14
hIgG4	–	NA	NA	3.05	NA	3.80	NA	NA	NA

NA, not applicable; –, not test.

### Assessment of the blocking properties of 405B8H3(D‐E)

**Fig. 3:**
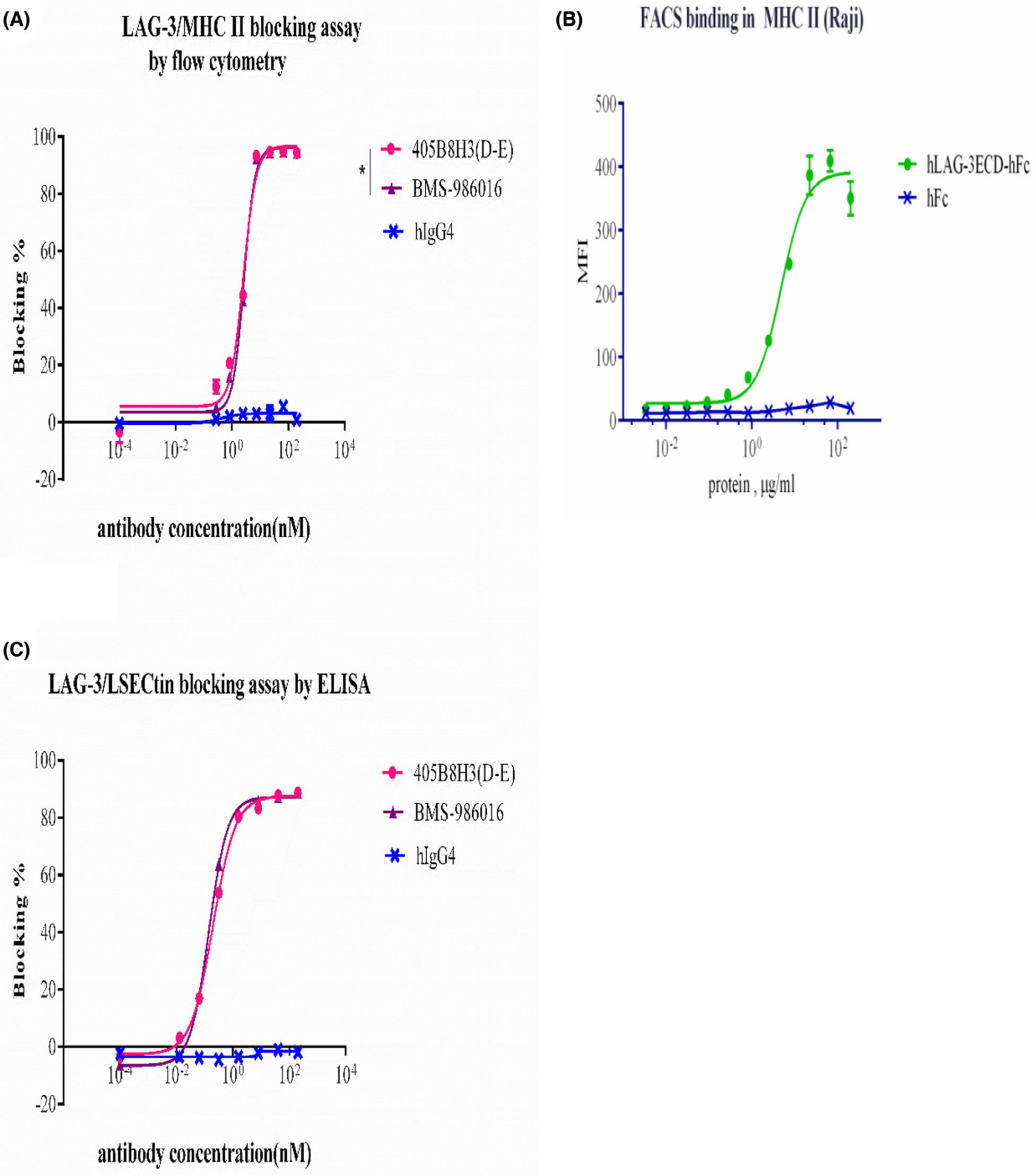
Blockade of LAG‐3 ligands' activity by different anti‐LAG‐3 antibodies. (A) Raji‐MHC‐II cells were incubated with hLAG‐3ECD‐hFc and blocked with either 405B8H3(D‐E), BMS‐986016, or a negative control (hIgG4) at different concentrations. A secondary Alexa Fluor 488‐conjugated antibody was used for detection by flow cytometry. The mean fluorescence intensity is shown as the mean ± SEM, calculated from experimental duplicates. Statistics were performed using two‐way ANOVA analysis; *P < 0.05. (B) Raji‐MHC‐II cells were seeded onto a 96‐well plate. The hLAG‐3ECD‐hFc and hFc protein were, respectively, added into harvested Raji cells. A secondary Alexa Fluor 488‐conjugated antibody was used for detection by flow cytometry. The mean fluorescence intensity is shown as the mean ± SEM, calculated from experimental duplicates. (C) 405B8H3(D‐E), BMS‐986016, and a negative control hIgG4 were assessed for their ability to block LAG‐3/LSECtin interaction by ELISA with hLAG‐3ECD‐hFc‐coated plates co‐incubated with either of the two anti‐LAG‐3 antibodies or a negative control (hIgG4) at different concentrations, and with LSECtin His tag (50 μL/well) at 37 °C for 2 h. A secondary HRP‐labeled antibody and substrate were used for detection. Absorbance data are shown as mean ± SEM calculated from experimental duplicates.

The IC50 values of 405B8H3(D‐E) and BMS‐986016 were compared in an inhibition assay testing their ability to interfere with LAG‐3/MHC‐II and LAG‐3/LSECtin interactions. Upon binding of LAG‐3 to MHC‐II, inhibitory signals are transmitted through LAG‐3 cytoplasmic domain [27] and facilitate tumor escape [28]. In addition, MHC‐II expression by melanoma may recruit tumor‐specific CD4+ T cells that produce TNF‐α, leading to a reduced CD8+ T‐cell response [29]. Therefore, the capacity of anti‐LAG‐3 biologicals to block LAG‐3/MHC‐II interaction is an important parameter. First, the blocking properties of the two previously mentioned LAG‐3 antibodies were compared by FACS. In MHC‐II (Raji) binding assay, Raji cells were derived from a Burkitt‐like lymphoma that carries low‐avidity IgG Fc receptors on its surface, rendering the binding of hLAG‐3ECD‐hFc to Raji cells through Fc receptors unlikely. Further strengthening this point, MHC‐II could bind hLAG‐3ECD‐hFc fusion protein in a dose‐dependent manner, but not hFc. The IC50 of 405B8H3(D‐E) (2.55 nm) was similar to that of BMS‐986016 (2.66 nm; Fig. 3A). LSECtin, another ligand of LAG‐3, is expressed in liver and melanoma cells; thus, anti‐LAG‐3 antibodies could be used to reactivate NK and CD8+ T‐cell function in these environments [15]. Next, we evaluated the ability of 405B8H3(D‐E) to effectively inhibit the binding of human LAG‐3 to LSECtin by ELISA blocking assay. The IC50 of 405B8H3(D‐E) (0.20 nm) was similar to that of BMS‐986016 (0.14 nm; Fig. 3C). In this assay, the IC50 value of 405B8H3(D‐E) (IC50 = 0.20 nm) was not significant different from that of BMS‐986016 (IC50 = 0.14 nm; Fig. 3B).

### 405B8H3(D‐E) increases IL‐2 production upon SEB stimulation

**Fig. 4:**
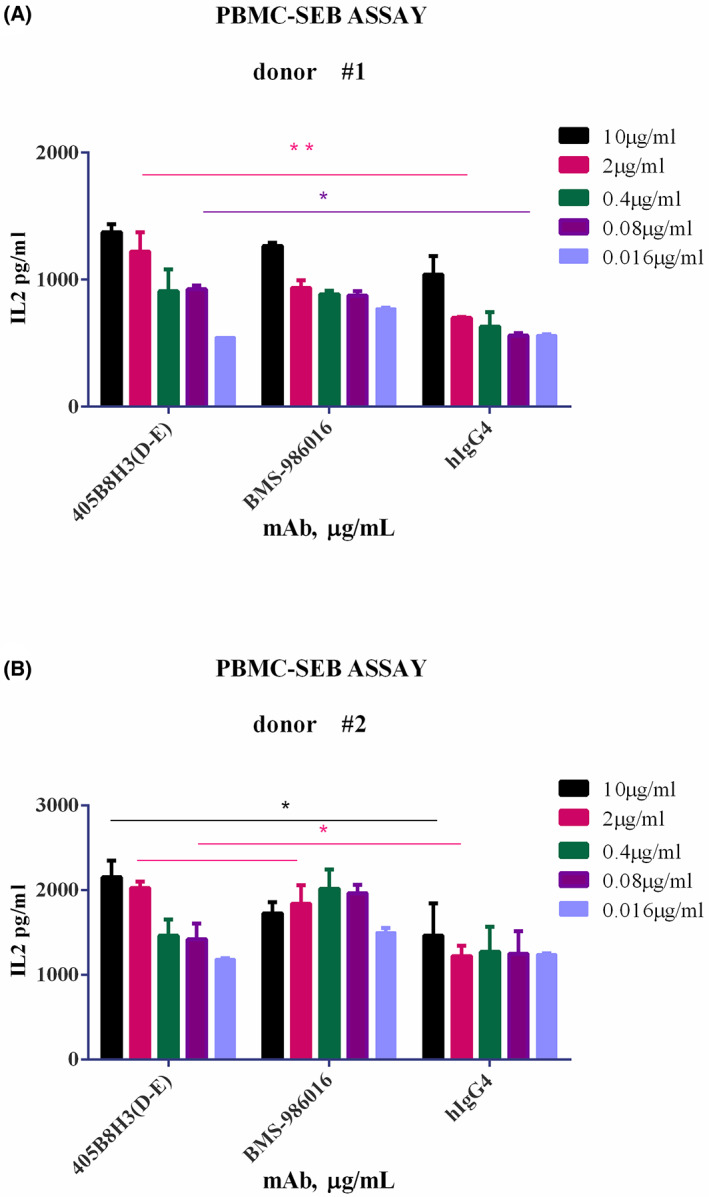
Assessment of IL‐2 release upon anti‐LAG‐3 antibody exposure of Staphylococcal enterotoxin B (SEB)‐stimulated human peripheral blood monocytes (PBMC). PBMC (1 × 10^5^ cells) from two different healthy donors; donor #1 (A), donor #2 (B) were activated by plate‐bound SEB (100 ng mL^−1^) and anti‐LAG‐3 antibodies at concentrations ranging from 0.016 to 10 μg mL^−1^. IL‐2 secretion was determined by ELISA. For each donor, this assay was performed in duplicate, and the data are shown as the mean ± SEM. At both concentrations of 10 and 2 μg·mL^−1^, the IL‐2 secretion stimulated by 405B8H3(D‐E) was superior to that obtained with BMS‐986016 (A, B). The differences in IL‐2 secretion between groups were analyzed using two‐way ANOVA analysis; *P < 0.05, **P < 0.01.

The expression of LAG‐3 and its strong affinity for MHC‐II are significantly upregulated under inflammatory conditions. IL‐2 controls CD4+ T‐cell proliferation and enhances their sensitivity to Treg inhibition [30]. Both 405B8H3(D‐E) and BMS‐986016 increased IL‐2 release by PBMC freshly isolated from three healthy donors exposed to the superantigen SEB, and this effect of 405B8H3(D‐E) appeared to be dose‐dependent in two donors (Fig. 4). At both concentrations of 10 and 2 μg·mL^−1^, the IL‐2 secretion stimulated by 405B8H3(D‐E) was superior to that obtained with BMS‐986016 (Fig. 4A,B). The differences in IL‐2 secretion between groups were analyzed using two‐way ANOVA analysis; *P < 0.05, **P < 0.01. According to its biochemical behavior and functional characterization, 405B8H3(D‐E) is a potentially effective therapeutic antibody worthy of further investigation.

### Assessment of antitumor activity *in vivo*

**Fig. 5:**
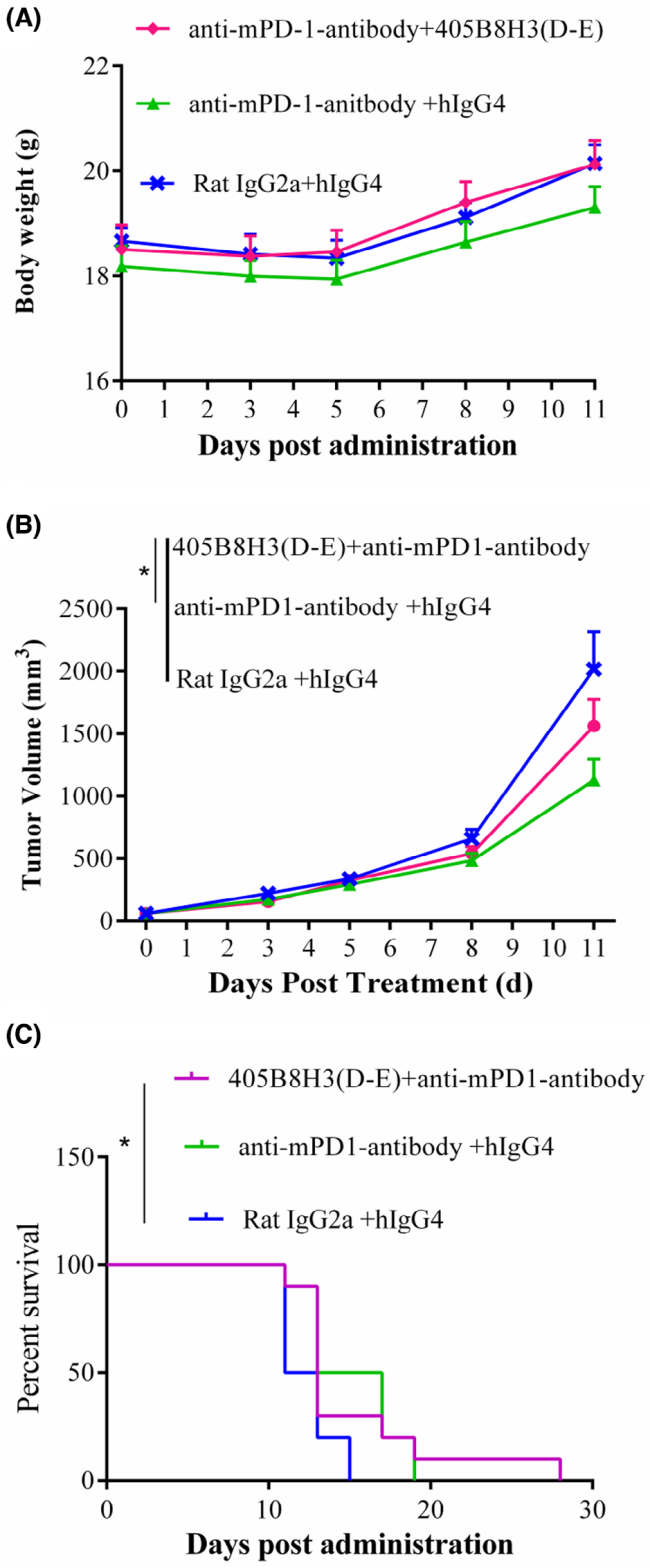
Study of 405B8H3(D‐E) efficacy *in vivo*. MC38 tumor‐bearing human LAG‐3 knocked‐in mice with measurable tumors were randomized and treated by intraperitoneal injection with 405B8H3(D‐E) combined with anti‐mPD1‐antibody [405B8H3(D‐E) + anti‐mPD‐1], anti‐mPD1‐antibody alone [anti‐mPD‐1‐antibody + hIgG4], or control hIgG4 [Rat IgG2a + hIgG4] on days 0, 3, 7, and 11. (A) Body weight; bars represent means ± SEM. (B) Tumor size; the statistical significance was determined at endpoint values by two‐way ANOVA; *P < 0.05. (C) Survival curve of the mice with tumor volume reached > 2000 mm^3^ as endpoint; the log‐rank test is shown;*P < 0.05. The entire study was terminated on D28, when the last mouse reached the endpoint.

Matsuzaki *et al*. [9] demonstrated that double blockade of LAG‐3 and PD‐1 pathways in human ovarian cancer improved the recovery of T‐cells' effector functions. We next evaluated the antitumor activity of the newly developed chimeric anti‐LAG‐3 antibody 405B8H3(D‐E) combined with anti‐mPD‐1 antibody in a tumor model consisting of C57BL/6 mice carrying a human LAG‐3 knock‐in inoculated subcutaneously with the MC38 tumor cell line (Table 2). During the treatment period, no significant body weight loss or visible adverse effects were observed in any of the treatment groups (Fig. 5A). Anti‐hLAG‐3405B8H3(D‐E) (10 mg·kg^−1^) combined with anti‐mPD‐1 antibody (10 mg·kg^−1^) efficiently reduced tumor growth compared with treatment with an isotype control, but anti‐mPD‐1 antibody alone showed superior efficacy in limiting tumor growth compared with combined therapy (Fig. 5B). The statistical significance was determined at endpoint values by two‐way ANOVA; *P < 0.05. However, anti‐hLAG‐3405B8H3(D‐E) combined with anti‐mPD‐1 seemed likely to improve mouse survival compared with treatment with anti‐mPD‐1 alone (Fig. 5C). Time‐to‐endpoint Kaplan–Meier survival analysis was performed using a tumor volume of 2000 mm^3^ as endpoint (Fig. 5C).

**Table 2:** Efficacy of anti‐human LAG‐3 antibodies combined anti‐mPD1‐antibody in MC38 tumor‐bearing human LAG‐3 knock‐in mice.

Tumor volume (mm^3^)					
Group	*N*	Mean ± SEM/grouping	Mean ± SEM/days postadministration	Dose (mpk)	Schedule
405B8H3(D‐E) + anti‐mPD‐1 antibody	10	63.22 ± 2.72/D0	1564.28 ± 211.72/D11	10 mg·kg^−1^ + 10 mg·kg^−1^	i.p. BWi
Anti‐mPD‐1 antibody + hIgG4 10	10	63.44 ± 2.89/D0	1130.8 ± 167.01/D11	10 mg·kg^−1^ + 10 mg·kg^−1^	i.p. BWi
RatIgG2a + hIg G4	10	63.67 ± 2.87/D0	2017.09 ± 298.14/D11	10 mg·kg^−1^ + 10 mg·kg^−1^	i.p. BWi

Mice bearing MC38 tumors were randomized into three groups (*n* = 10 per group), and candidate antibodies were administered intraperitoneally at a dose of 10 mg·kg^−1^ + 10 mg·kg ^−1^ (mpk). Data are shown as the mean ± SEM; BWi, body weight of a mouse on a specific day.

## Discussion

As an inhibitory coreceptor, LAG‐3 (CD223) inhibits T‐cell activation and cytokine secretion to ensure immune homeostasis [31]. LAG‐3 interacts with MHC‐II to prevent its binding to the T‐cell receptor (TCR) and CD4, thereby hindering TCR signaling during immune responses [32]. The LAG‐3/MHC‐II interaction may also serve as a bidirectional inhibitory signal co‐joined by tumor and immune cells [28]. Cancer immunotherapy is a rapidly growing field, and numerous checkpoint molecule antagonists have been developed in the treatment for solid tumors. Combining cytotoxic T lymphocyte antigen‐4 (CTLA‐4) and programmed cell death 1 (PD‐1) blockade has improved treatment efficacy against metastatic melanoma despite its high toxicity and severe adverse effects in a high proportion of patients [33]. Other potential checkpoint molecule targets are getting more attention. Among these, LAG‐3 is currently being investigated as a novel checkpoint therapeutical target. Here, we report the development of 405B8H3(D‐E), a chimeric IgG4 antibody with high affinity for LAG‐3 able to block LAG‐3/MHC‐II and LAG‐3/LSECtin interactions and promote IL‐2 secretion *in vitro*. Blockade of the interactions between LAG‐3 and its ligands seems crucial for its inhibitory function.

The inhibition of two immune checkpoint molecules, LAG‐3 and PD‐1, exhibited more benefit than the inhibition of PD‐1 alone in patients [34]. In MC38 tumor‐bearing hLAG‐3 knock‐in mice, 405B8H3(D‐E) combined with anti‐mPD‐1 antibody prolonged survival compared with anti‐mPD‐1 antibody alone and control treatment. Hotspot DNA sequences are frequently located in the CDRs and are strategic to increase antibody affinity [35]. However, alteration of the highly conserved primary and higher‐order structures, or commonly recognized degradation hot spots, such as Asp isomerization in CDRs, an important chemical degradation pathway for recombinant mAbs, may decrease or lead to a loss of affinity. Therefore, a careful examination of protein stability is necessary [20]. According to the protein sequence, the Asp‐Gly (DG) motif was laying in the CDR2 of the candidate murine anti‐LAG‐3 antibody light chain. Because of its better avidity, we preferred the EG mutated variant antibody to SG mutated variant antibody.

Moreover, 405B8H3(D‐E) exhibited higher cross‐reactivity with cynomolgus monkey LAG‐3 than BMS‐986016, suggesting that 405B8H3(D‐E) may have wider applications and could be used in primates for *in vivo* preclinical toxicology and pharmacokinetics research. Therefore, 405B8H3(D‐E) is an optional antagonist for antitumor cells (Tables 1 and 2). It was interesting that the combination of BMS‐986016 (anti‐LAG‐3) and nivolumab (anti‐PD‐1) demonstrated an exciting initial effect in melanoma patients who were refractory to previous anti‐PD‐1/PD‐L1 therapies (NCT01968109; www.clinicaltrials.gov). The results we obtained with 405B8H3(D‐E) used as combined treatment with anti‐mPD‐1 antibody in a mouse tumor model, and the overall properties of this antibody suggested that 405B8H3(D‐E) would be an optional component for combined therapy, while the combination of 405B8H3(D‐E) with an anti‐PD‐1 mAb does not seem to significantly decrease the tumor size in MC38 model, compared with monotherapy. To clarify the antitumor effect of combined therapy, we will plan to try Human PD‐1/Human LAG‐3 double knock‐in mice tumor model [36]. In conclusion, the growing body of clinical data demonstrating the efficacy of checkpoint inhibitor therapy and the properties of 405B8H3(D‐E) support that this new antibody is a potential candidate for cancer immunotherapy.

## Conflict of interest

TTCY, NS, and YW are current employees of ChemPartner. BQ is a current employee of Jiangsu Huaiyu Pharmaceutical. The patent pertaining to the results presented in this paper has been filed by PharmaExplorer (Publication Number CN 110343178 A). The other author declares no conflict of interest.

## Author contributions

NS designed the experiments, performed the experiments, and analyzed the data; XL edited the manuscript; SR, BQ, and YW reviewed the manuscript; and TTCY supervised the project.

## Data Availability

The data of this study are available from the corresponding author upon reasonable request.
